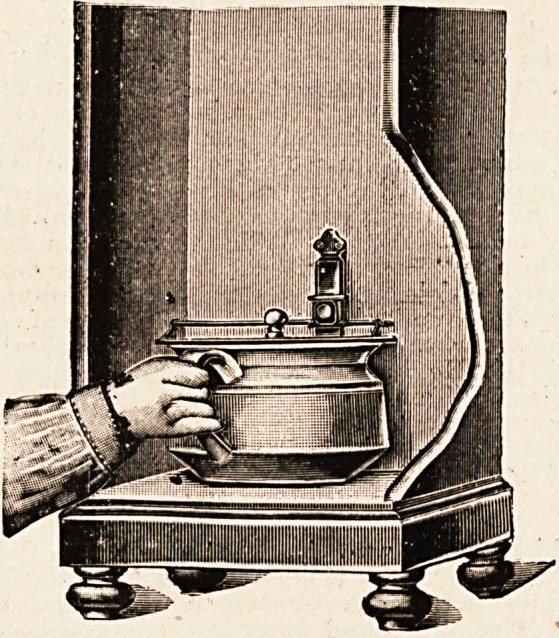# New Appliances and Things Medical

**Published:** 1907-08-03

**Authors:** 


### NEW APPLIANCES AND THINGS MEDICAL.

[We shall be glad to receive at our Office, 28 & 29 Southampton Street, Strand, London, W.C., from the manufacturers, specimens of all new preparations and appliances which may be brought out from time to time.]

### THE "AUTOLID" SANITARY COVER.

(The Sanitary Appliances Co., Greenwich, London, S.E.)

This is a simple yet ingenious cover for bedroom toilet utensils which can be fixed in any position. The appended illustration shows its adaptation to ordinary purposes better than would any lengthened description. It possesses the advantages of being practically unbreakable, as it is of solid brass, and of being very easily cleaned and sterilised.

### THE EQUIPOISE BED.

(Equipoise Co., Ltd., Ashford).

The bedstead manufactured by the Equipoise Company is one which will commend itself to everyone who appreciates anything that can materially lighten the task of nursing a bed-ridden patient in cases where hospital conveniences and the facilities for frequent changing of position by ordinary methods are not available. We have had several opportunities of seeing the bedstead in actual use, and on each occasion it was noticeable that nurse, as well as patient, was spontaneously loud in its praise. At first sight it must be admitted that the appliance seems unwieldy, even cumbrous, but when one comes to work it, and to realise how easily and with how little effort on the part of the attendant, almost any conceivable change of position can be effected, without disturbing the patient, the invaluable assistance of such a convenience in nursing becomes apparent at once. No rests, cushions, straps, headpieces, or similar aids to maintaining any position are required. Every position is easily obtained by working a simple catch which in most cases the patient can work himself, without any assistance from the nurse. There is no complicated mechanism to get out of order; the whole principle of the bedstead is simplicity combined with strength, and from what we have seen of it such a bedstead seems likely to last more than the proverbial lifetime.

The same company supplies chairs, lounges, sofas, and couches, made on the same principle, and all, without exception, beautifully upholstered and finished. An illustrated booklet, giving full particulars of the various positions, etc., can be obtained on application to the manager.

## Figures and Tables

**Figure f1:**